# “I’m walking on eggshells”: challenges faced by mothers with breast cancer in interacting with adolescent daughters

**DOI:** 10.1186/s12905-022-01872-1

**Published:** 2022-09-20

**Authors:** Pingting Zhu, Qiaoying Ji, Xinyi Liu, Ting Xu, Qiwei Wu, Yuejuan Wang, Xu Gao, Ziheng Zhou

**Affiliations:** 1 School of Nursing, Yangzhou University, 136 Jiangyang Middle Road, Yangzhou, 225009 China; 2 Jiangsu Key Laboratory of Zoonosis, Yangzhou, China; 3 Clinical Medical College, Yangzhou University, Yangzhou, China

**Keywords:** Breast cancer, Mothers, Adolescent daughters, Relationships, Women’s health, Qualitative

## Abstract

**Background:**

With breast cancer becoming the most diagnosed cancer in the world, the number of breast cancer-afflicted mothers with adolescent daughters is also rising. Further, adolescent daughters’ mothers serve as role models for in identity formation processes, especially concerning gender and sexuality. Nevertheless, breast cancer threats mother’s health, including such a key symbol of her womanhood—the breast—which may adversely affect the development of an adolescent daughter’s own sense of personal identity and womanhood. However, few researchers and practitioners have paid attention to mother-daughter interactions in the context of breast cancer. Therefore, this study aimed to uncover the nuances of the interactive challenges with adolescent daughters from breast cancer-afflicted mothers’ perspective.

**Methods:**

We conducted a qualitative study following the sample saturation principle, collecting data through semi-structured interviews with 21 breast cancer patients who met the inclusion criteria. We utilized thematic analysis and partially integrated the Foucauldian discourse approach to analyze the data.

**Results:**

Three major themes emerged from the data: (1) mothers are lost in chaos (inability to handle the shock of cancer, feelings of powerlessness about the uncertainty of their life span, and confusion about how to respond to daughter’s curiosity); (2) mothers struggle to maintain balance (torn between protecting daughters and letting them be independent, and making a tough choice between being a mother or a patient); and (3) mothers are immersed in guilt (increasing daughters’ risk of cancer, influencing daughters’ development, and imposing burdens on daughters).

**Conclusions:**

Our research explored the interactive experience of breast cancer-afflicted mothers and adolescent daughters. The insights uncovered by this study will help mothers enhance interaction with their daughters and assist health practitioners in devising interventions.

**Supplementary Information:**

The online version contains supplementary material available at 10.1186/s12905-022-01872-1.

## Background

Breast cancer has become the most diagnosed cancer in the world [1]. In 2020, women aged 15 to 44 accounted for 33.5% of breast cancer cases, which means that a large number of women with breast cancer may have children younger than 18 years [2]. These children rely on their mother in daily life and may experience difficulties in coping with their mother’s diagnosis and treatment of breast cancer [3, 4]. Given the volatility of the stages of adolescence, adolescents (especially girls) are at heightened risk of being impacted negatively impacted by their mothers’ illnesses [5, 6]. This is because girls can become more involved in the household duties, struggle to become independent, and are more sensitive to their mothers’ illness-related stress [7]. Importantly, breast cancer is different from other oncological diseases and plays a unique role in mother-daughter relationships. First, mother’s illness increases daughters’ risk of breast cancer [8]. As shown in Chan’s research, more than half daughters whose mothers were diagnosed with breast cancer reported elevated levels of worry about breast cancer; that is, their mother’s illness heightened their personal sense of vulnerability to the disease [9]. Additionally, adolescent daughters are actively forming their gender femininity and sexuality [10]. Breast cancer threatens their mother’s health, including a key symbol of her womanhood—the breast [11]—which may disrupt adolescent girls’ confidence and understanding of their own developing bodies [12]. Furthermore, mothers serve as a primary socializing figure among adolescent daughters [13]; thus, their breast cancer can influence the development of adolescent daughters’ own sense of personal identity and womanhood [14]. Therefore, mothers with breast cancer face their adolescent daughter’s distress and fear in addition to experiencing the physical and psychological symptoms of breast cancer [15].

However, researchers and practitioners have focused mainly on marital interactions, while there are few studies on mother-daughter interactions in the context of breast cancer [16, 17]. This may be because the relationship between mothers and daughters is by nature intergenerational, which embodies unique challenges [18]. Although scarce, existing research suggests that breast cancer-afflicted mothers and their daughters report shared changes on many levels and that emotional problems are mutual [19]. Higher daughter anxiety has been linked to higher maternal anxiety and poorer family communication, and higher daughter breast cancer-specific distress is associated with higher maternal breast cancer-specific distress [20, 21]. These psychological effects can even extend further and result in physiological changes (e.g., impaired immunological functioning and increased stress hormones) for both mothers and their daughters [22]. However, the existing literature on the nuances of challenges in the interactions between breast cancer-afflicted mothers and their adolescent daughters has been limited. In our research, we sought to better understand the interactive challenges faced by breast cancer-afflicted mothers and their daughters from the former’s perspective.

It is important to explore the latent challenges and complicate interactions among mothers with breast cancer. As such, we integrate Foucauldian discourse approach [23] into our analysis when we familiar with the interview transcripts. This is utilized to examine discourses, revealing complex and conflicting perspectives in the interactions between breast cancer-afflicted mothers and their adolescent daughters. Identifying major themes in these interactions may assist health practitioners in developing helpful interventions in this context.

## Methods

### Study design and participants

To explore and better understand the subjective experiences of mothers with breast cancer, we collected data through a phenomenological research design based on a qualitative research approach [24, 25]. This is a qualitative research method that seeks to understand complex phenomena through the participants’ lived experience, meaning, and perspectives [26]. We also needed to recruit participants with specific knowledge and lived experience, who were able to articulate their views and perceptions. Therefore, we used purposeful sampling with maximum diversity to extract breast cancer-afflicted mothers with adolescent daughters [27]. The inclusion criteria for participants consisted of the followings: (1) female patients; (2) currently raising daughters between ages 13 and 18; (3) normal cognitive and language expression abilities and no verbal communication barriers; and (4) sufficiently informed of the study’s purpose and content, voluntarily choosing to participate. The exclusion criteria were inability to complete the interview. Recruitment was started with convenience sampling and reviewed demographics after ten interviews; then we purposefully sampled more breast cancer-afflicted mothers with diversity in age, education, disease stage and time of diagnosis. Interviews were conducted from November 2020 to September 2021, and the data analysis was performed concurrently with data collection. Saturation should be confirmed only after no new themes were found in three consecutive interviews [28]. A total of 26 mothers were invited to participate in the study, but 5 eligible women refused to participate due to being too busy or prefer not to talk about their families. Ultimately, 21 breast cancer mothers were interviewed. Participants’ demographic characteristics were shown in (Table 1).

**Table 1:** Sample characteristics (N = 21)

Characteristic	N (%)
Age (years)	
Mean (range)	40.33 (31–49)
Diagnosis of breast cancer (months)	
Mean (range)	8.25 (2–23)
Highest educational level	
Primary educational level	9 (42.9%)
Ordinary level	7 (33.3%)
Advance level	5 (23.8%)
Disease stage	
I	2 (9.5%)
II	7 (33.3%)
III	9 (42.8%)
IV	3 (14.3%)

### Procedure

Before initiating the study, we obtained human subjects approval from the Ethics Committee of School of Nursing of Yangzhou University, with the IR code: YZUHL2021006. The potential participants were identified by the oncologists through a review of clinic lists and medical records in several hospitals. Subsequently, the oncologist introduced the interested mothers to the Principle Investigator (PI) to confirm whether they met the inclusion criteria. Then, we sent a recruitment letter to the mothers who met the inclusion criteria to inform them about the study and asked them to contact the PI within one week if they were willing to participate. The interested mothers signed and returned the consent form to the PI, after which the PI scheduled a face-to-face interview. Before the interview, researchers participated in qualitative research training courses and watched and analyzed excellent interviews repeatedly to systematically learn theoretical knowledge and interview skills. The pre-interview outline was constructed by reviewing the relevant literature on communication between parents diagnosed with cancer and their children. After the pre-interview of two participants, the interview outline was adjusted, and then two experts in the field of cancer were invited to make two rounds of revisions to form a formal interview outline (Table 2). Prior to the interview, the researchers established a relationship of mutual trust with the participants through greetings. QYJ and XYL conducted the interviews in a private room in the hospital according to the interview outline, and adjusted according to the actual situation and need. During the interview, researchers used various communication skills, such as active listening, nonverbal cues and tolerating silences. Interviews were audio-recorded with permission, and lasted 25 to 72 min (median 45 min).

**Table 2:** Interview questions

1. How do you interact with your daughter(s)? Can you describe the details of your relationship?
2. What is the impact of breast cancer diagnosis on your interaction with your daughter(s)?
3. Could you tell me about your inner activities when interacting with your daughter(s) after the diagnosis?
4. What impressed you during your interaction with your daughter(s) after the diagnosis?
5. Is there anything you want to talk about that I didn’t ask?

### Data analysis

A professional transcriptionist transcribed the recorded interviews within 24 h and in strict accordance with the requirements of data system analysis, and the PI verified for the transcripts for accuracy. The Braun Clarke Thematic Analysis method and the Foucauldian discourse analysis were used in the data analysis process [29, 30]. At the beginning of the data analysis process, all researchers repeatedly read the interview records line by line and paragraph by paragraph to become familiar with the contents of the data. Authors PTZ and QYJ initially inductively coded four interviews, compared notes, and then coded four more interviews and established a coding structure agreed upon by PTZ, QYJ and XYL. Then the research team double coded the remaining interviews and organized the coded data into themes. In all cases, we reached at least 80% agreement in assigning codes between two researchers. Disagreements were resolved by further discussion among the researchers. Themes emerged from the data rather than deductively obtaining them from pre-existing themes in the literature, and each theme reflected a trend within the data set. Through an iterative process, we added new codes to the coding structure as they emerged in the analysis. We used NVivo to organize the data. And as a supplementary, Foucauldian discourse analysis was used in reading and interpreting the text when familiar with the transcript. It was used to critically with the complexities of discourse and making sense of the emergence of the smallest of statements [31]. After discussing and comparing the data, the researchers reached consensus. Three themes and eight subthemes emerged (Additional file 1). We also used several strategies to ensure the data’s reliability. First, we sent the transcripts to the corresponding participants to confirm the transcribed content’s accuracy. Second, the researchers held regular, continuing discussions to verify the appropriateness of the conceptual meanings and terminology. Third, we maintained an audit trail to ensure that all analysis steps could be traced back to the original interviews. Finally, all research results were derived via the entire research team’s consensus.

## Result

### Theme 1 mothers are lost in chaos

Most interviewees expressed experiences of feeling trapped in chaos when they were newly diagnosed with breast cancer, providing various example. We divided this theme into three subthemes: (1) inability to handle the shock of cancer, (2) feelings of powerlessness about the uncertainty of their life span, and (3) confusion about how to respond to daughter’s curiosity.

#### Inability to handle the shock of cancer

Mothers expressed that when they received the diagnosis of breast cancer, their family began a new trajectory. They tend to describe that moment as a bombardment and blindsided, when received information about the diagnosis and the treatment options. One mother who had not previously been familiar with breast cancer indicated:

(P2) At that time, I didn’t know how serious my illness was, I felt unsure about how to approach the issue. I’m confused!

Other mothers in our study also indicated that they tended to prioritize dealing with devastating crises (such as diagnosis/treatment and marital crisis) under the shock of cancer and overlook the mother-daughter relationship. They disclosed that their adolescent daughters were acutely aware of abrupt changes in the family atmosphere, for which mothers felt unprepared.

(P14) Of course, for a while after my diagnosis I was depressed about breast cancer…I don’t know how clear I am about the results and don’t have the energy to think about my relationship with my daughter.

#### Feelings of powerlessness about the uncertainty of their life span

Participants in our study expressed that the diagnosis of breast cancer is typically a great shock to them, as they often associate cancer with death. Most mothers (N = 20) spoke about their likely limited life expectancy, and even if the newer treatments could extend their lives, they felt that it would never be enough time.

(P6) I’m confused about the future. I’m still adjusting myself. I don’t know how long I can stay with my daughter and how to face her. Maybe I still need time to tidy up myself.

When asked about inner activities when interacting with their daughters after diagnosis, mothers indicated that they always hoped to give their daughters life advice and prevent them from associating with the “wrong crowd”. However, breast cancer hinders mothers’ role because they cannot experience rich interactions with their daughters in such a limited time, as demonstrated in the following quote:

(P19) The thing I think that makes me most upset is her (my daughter). I could talk about me “I’m going to die one day.” But it’s her (my daughter) I think may need my help on her life path.

(P1) I just have this fear that … if something happens in the next couple years.

#### Confusion about how to respond to daughter’s curiosity

Mothers always describe themselves as the preferred source of sexual knowledge and discussion for adolescent daughters, they stated said that they were special to their daughters in some way. And they should protect her daughter from the effects of cancer not only cancer itself but also of mother’s deformed breast because of the special nature of breast cancer. This burden is reflected in the following quote:

(P2) I dare not let her see my breast because I had a mastectomy. I’m afraid it will cause psychological shadow to her (fear of deformed breast). I’m walking on eggshells!

Further, mothers tend to limit the discussion to safe topics in order not to influence daughters’ views on secondary sexual characteristics (e.g., breasts). Fourteen mothers mentioned that their daughters would consciously want to discuss breast issues with them, but they would choose to avoid out of uncertainty about breast cancer. As the seventeenth mother expresses:

(P2) I feel puzzled. Actually, I want to communicate with her (about breast cancer), but I don’t know how to do. My daughter is in puberty and secretly discusses with me that her breasts are changing…

Some mothers (N = 13) indicated that they hoped to communicate about their physical condition and treatment with their daughters, but they felt unsure about how to do so and how much information to share, making them feel confused. The practical and emotional challenges of communicating with daughter complicate their mother–daughter interactions; mothers were aware that they lacked evidence-based guidelines to manage and often were unsure how to proceed.

(P14) I think I should tell my daughter about my illness, but I don’t know how to tell her. I didn’t get any professional help in communicating with my daughter.

(P17) I hope to get guidance on how to communicate and make it easier for children to accept the facts. This requires children’s cooperation, and mainly depends on adults to guide.

### Theme 2 mothers struggle to maintain balance

A breast cancer diagnosis can have a devastating impact on both mothers and daughters. Mothers with breast cancer express that once they surmount the initial chaos, they strive to strike a delicate balance in their interactions with their daughters. We divided this theme into two subthemes: (1) torn between protecting daughters and letting them be independent and (2) making a tough choice between being a mother or a patient.

#### Torn between protecting daughters and letting them be independent

Mothers indicated that breast cancer affects the way they interact with their daughters, and they need to strive to strike a balance between protecting daughters and letting them be independent. On the one hand, they are worried that their daughters will have a psychological burden when they knew of the diagnosis. On the other hand, they felt they could not hide the diagnosis and acknowledged that the truth could make their daughters more independent, as echoed in the following:

(P8) I was afraid that telling her my illness would make her have a psychological burden, so I didn’t tell her at first, but later I found that she was more worried. She guessed every day and was very sad, so I told her after her enrollment and school choice were determined.

(P20) I think it’s better to show your down sides instead of trying to be strong all the time. I’ll need my daughter’s help, and I can make it with the courage she gives me. But… she doesn’t know what cancer is.

Some mothers (N = 9) expressed that factual explanation could simultaneously help their daughters become more independent and elicit emotional support from them (i.e., a mutually beneficial interaction). Most participants (N = 18) stated that they would tell their daughters the news when they could not hide it any longer, or they would observe their daughters’ reactions and decide whether to disclose the diagnosis at some point. Participants paid especially careful attention to their daughters’ behavior and language, which could be challenging.

(P13) I didn’t let my daughter go to the hospital before the treatment plan was decided. I don’t want to cause psychological shadow to her. But my hair will fall off after chemotherapy. I can’t hide it. I had to tell my daughter about my condition.

(P20) Telling her my condition may make her lose happiness, which is not what I want to see. I did not want to say too much to “scare them off”, yet I wanted to give my daughters knowledge to make her independent. I’m looking for a chance to let her understand my condition.

#### Making a tough choice between being a mother or a patient

Participants in this study reported that activities such as volunteering at their daughters’ schools, going on field trips, and driving daughters to and from school were difficult for them. They expressed the importance of doing everything that they had previously done. Nevertheless, this often required some adjustments for mothers with breast cancer in daily life. As some mothers described, even if they suffer from cancer and the painful treatment thereof, they still insisted on doing housework and helping maintain their daughter’s daily lives when their physical strength allows for it. They hoped to minimize the impact on their daughters’ study and lives and reduce the burden on others.

(P8) My daughter still asked me to send her to school and help her carry heavy things like I did when I was not ill, but sometimes my body can’t bear it. I was thinking about whether to show my daughter my wound, which might hit her, but let her know a little about my condition, maybe I wouldn’t be so tired.

(P12) I think if I were a single person without kids, this would be easier for me to deal with because the most difficult part is dealing with the kids. I still insist on housework and taking care of the children’s daily life even if I am ill.

Mothers explored that they need a long rest to recover from their exhausted physical strength due to the impact of breast cancer. During their illness, they make efforts to adjust and balance their roles as mother and patient, helping their daughters recognize that they will not be able to engage in certain activities for some time. Of course, some mothers (N = 4) believe that being a mother or a patient isn’t a though choice, because the role of mother takes precedence over other roles in their view.

### Theme 3 mothers are immersed in guilt

Our participants indicated that they had been immersed in guilt since their diagnosis. This guilt tends to occur for a long time and is distinct from the short phase of feeling trapped in chaos and struggling to maintain balance in the mother-daughter relationship. We divided this theme into three subthemes: (1) increasing daughters’ risk of cancer, (2) influencing daughters’ development, and (3) imposing burdens on daughters.

#### Increasing daughters’ risk of cancer

Mothers expressed feeling especially guilty when their daughters blamed them for having passed along the increased cancer risk. This resulted in dysfunctional mother-daughter interactions. Though mothers understood that their risk or disease was neither their fault and not within their control, they still felt responsible for their daughter’s disease risk. It’s a challenge for breast cancer-afflicted mothers to intellectually overcome such guilt and repair relationships with their daughters, as evidenced by the following quote:

(P16) I’m very worried that my daughter will get breast cancer. Because my breast cancer can cause my daughter to have a higher rate of disease than others. Sometimes, when I see my daughter, I feel guilty for her because I think her future trajectory will have some bad changes because of my breast cancer.

(P4) I’m very afraid that this disease will be passed on to my daughter. I’m guilty… it is difficult to overcome.

#### Influencing daughters’ development

Participants in our study expressed that the adverse reactions of postoperative and radiotherapy seriously affect their image, which lead to psychological problems such as low self-esteem and self-doubt among patients’ daughters sometimes. Further, the experience of losing their mother also would affect adolescent daughters’ emotions and personality. Mothers always take raising their daughters as their duty, while adolescent daughters are in an important stage of learning, they worry that their diseases will put pressure on their children and affect their learning. As breast cancer mothers in our interview has said, dealing with these interwoven emotions is a huge challenge:

(P6) I used to take her swimming, but I can’t to take her anymore. I afraid others looking at me with strange eyes. My illness may affect my daughter’s mental health and make her feel inferior. I don’t think I’m a good mother…

(P14) My daughter’s grades are always ahead in the class, but I haven’t helped her with her homework since I was ill. Her teacher found the mistake and criticized her and she was unhappy when she came home. I feel sad about it…

#### Imposing burdens on daughters

Mothers believe that breast cancer as a family genetic disease will affect their daughter’s marriageability, deterring potential partners because of the breast cancer genes that they carry. Some participants informed us that their adolescent daughters even remarked that she would never marry because they knew they would eventually have breast cancer, which places immense pressure on the mothers.

(P21) My daughter kind of holds me responsible both for her cancer and marriage. I’m very worried… my breast cancer is bad for my daughter to get married. Her partner may be estranged from her.

Additionally, the enormous financial burden to families is also an important reason why mothers with breast cancer feel guilty. Mothers expressed that their daughters who are at high risk for breast cancer must pay close attention to their diet and physical examination, which may impose additional burdens. When mothers were asked why they felt guilty, they stated their illness had reduced the quality of life of the entire family. This effects the mother-daughter relationship, as demonstrated in the following quotes:

(P10) The cost of physical examination in the coming decades is also a great expense. Moreover, my daughter is more limited than other girls of the same age in terms of diet, living habits and even mate selection, which is unfair to her…

(P12) Our savings must be used for treatment and bear the education and living expenses of our two children. Bur now, I spend most of my money on cancer treatment, there will be no way to guarantee the future quality of life of my children.

## Discussion

Previous research has demonstrated that adolescence is a crucial phase in which daughters identify with their mothers and then move toward independence [10, 32]. Adolescent daughters tend to experienced more emotional distress compared to sons because of heredity and shared changes with their mother on many levels [33]. However, few studies have explored the nuances of challenges among breast cancer-afflicted mothers and their adolescent daughters. Our study reveals the experience of breast cancer-afflicted mothers “walking on eggshells” when interacting with adolescent daughters, which may enhance health practitioners’ understanding of mother–daughter dynamics and offer some guidance.

Lundquist et al. found that the most difficult aspect of being a young woman with breast cancer is being a mother [34]. There are many conflicts between adolescent daughters and their mothers, and breast cancer puts additional strain on this relationship [35]. As our participants disclosed, the uncertainty of information and relationships leads to difficulties in getting along with their daughters. Mothers must address their inner confusion before they can address relationship problems with their daughters, but this is particularly difficult given the physical and psychological issues caused by breast cancer (e.g., lymphedema or depression) [36]. Patients commonly experience information bombardment immediately after receiving a breast cancer diagnosis. They cannot consider the extent to which cancer will harm their body or their future life will be affected. As some research results show, most mothers will avoid interactions with their daughters in the early stages of the disease [37]. Until they resolve their inner doubts or can no longer hide the truth, they will only talk with their daughters about clearly observable consequences (e.g., hospitalization, exhaustion/fatigue, and hair loss). During this period, they will be selective in interacting with their daughters, which will hinder the development of mother daughter relationship and bring challenges to mothers suffering from breast cancer. Although avoidance is an understandable reaction and may help reduce or prevent distress in the short run, in the long run it often leading to even greater distress (e.g., decreased quality of life, elevated distress and anxious) [38, 39] Therefore, mothers should adjust their attitudes toward the disease, combine direct communicative approaches (e.g., being honest and open, asking questions) and more indirect strategies (e.g., using humor, sugarcoating, finding common ground) is beneficial to improve communication with adolescent daughters [40, 41].

Adolescent daughters of breast cancer-afflicted mothers are in a life in which identity development is key to their health, and they are experiencing change with respect to secondary sexual characteristics [7]. Their maturation and struggles with their sense of self will involve their mothers’ cancer experience and their own disease risk because mothers are in a unique position to provide emotional validation, health education, and secondary sexual characteristics guidance [14]. As our results shown, breast cancer-afflicted mothers tend to experience great difficulty in assisting with their adolescent daughters’ physical development problems, which can attribute to breast cancer’s destruction of mothers’ bodily integrity and potentially placing their daughters at higher risk for cancer. Notably, our participants study may be more introverted due to the influence of Confucianism, making it more difficult to discuss the development of secondary sexual characteristics with their adolescent daughters [42, 43]. This issue has rarely been reported in studies in other countries, which may be caused by cultural differences. Of course, promoting the interaction between breast cancer-afflicted mothers and their adolescent daughters is also an issue worthy of common concern for worldwide health practitioners. Our qualitative research reveals the challenges faced by breast cancer-afflicted mothers in the process of interacting with adolescent daughters, which provides a practical basis for the formulation of future interventions. According to the information disclosed by the participants in our study, it is worthy of further research to build an effective platform for mothers and daughters to promote communication while learning about health promotion together.

Consistent with Tavares’s research [3], we found that mothers are under tremendous pressure and feel guilty after suffering from breast cancer. They are always afraid that their diseases and treatment will negatively impact their adolescent daughters’ psychology, learning, and daily life. They even worry about the impact of their disease on their daughter’s marriageability and economic situation. Incorporating a life outside of cancer into mother-daughter relationship may help mothers mitigate this outcome [44]. And aware of what they think is responsible for having developed cancer can also be helpful to open an important internal dialog about guilt and self-forgiveness [45]. Furthermore, some mothers in our study expressed that their concerns and feelings of powerlessness about their mortality, reemergence of their cancer, and not seeing their daughters grow up hindered their interaction with their daughters. Planning for their future life in a positive way, focusing on what they can do to improve their family’s health and quality of life may help some mothers interact with their daughters in a more relaxed fashion [46].

In general, our findings reveal the challenges faced by mothers with breast cancer in interacting with adolescent daughters. Although our current study only included mothers of adolescent girls, our findings might apply to the relationships of breast cancer-afflicted mothers and daughters of other age groups. Adolescence is a special stage that bridges childhood and maturity [47, 48]. However, at present only a few health professionals are focusing on the important relationship between breast cancer-afflicted mothers and adolescent daughters [19, 49]. Our findings can serve as a window for health practitioners to understand the complexity of the relationship between breast cancer-afflicted mothers and their adolescent daughters, and inform needs assessments for resources to be provided to these individuals.

## Limitations

This study’s findings are limited in their scope of interpretation by the fact that this sample of women was small and limited to the mothers’ perspectives. Future studies could better explain the psychological status, family function, communication, and other related factors of breast cancer patients and their adolescent daughters through quantitative research. Such studies could further explore the intervention measures for breast cancer-afflicted who are raising their minor children. In addition, future research should also capture daughters’ experiences to enhance mother-daughter interactions.

## Conclusion

Our findings explored the interactive experience of breast cancer-afflicted mothers and their daughters, revealing the nuanced challenges in such interactions from mother’ perspective. One such challenge is mothers’ feeling lost in chaos when they are diagnosed with breast cancer and their struggle to maintain balance. Further, mothers are immersed in long-term guilt when interacting with their daughters. We hope that these findings will help inform strategies through which mothers can enhance interactions with their daughters and learn to cope with cancer risks in a healthier manner, as well as assist health practitioners in devising targeted interventions.

## Supplementary Information

**Additional file 1.** The themes and frequency.

## Data Availability

Data used in this paper are not publicly available due to the sensitive nature of the topic and the risk of breaching confidentiality, but are available from the corresponding author on reasonable request.
