# Supplementary material for: “I’m walking on eggshells”: challenges faced by mothers with breast cancer in interacting with adolescent daughters
Source: BMC Womens Health. 2022 Sep 20;22:385. doi: 10.1186/s12905-022-01872-1 (PMC9490998; doi:10.1186/s12905-022-01872-1)
Supplement: Supplementary file 1 — Additional file 1. The themes and frequency. [file 12905_2022_1872_MOESM1_ESM.docx]

**Supplementary material**

The themes and frequency

| **Themes** | **Sub-themes** | **Number of participants(frequency)** |
| --- | --- | --- |
| 1. Mothers are lost in chaos | 1.1. Inability to handle the shock of cancer | 19 (90.5%) |
|  | 1.2. Feelings of powerlessness about the uncertainty of their life span | 20 (95.2%) |
|  | 1.3. Confusion about how to respond to daughter's curiosity | 21 (100.0%) |
| 2. Mothers struggle to maintain balance | 2.1. Torn between protecting daughter and letting her be independent | 19 (90.5%) |
|  | 2.2. Making a tough choice between being a mother or a patient | 15 (71.4%) |
| 3. Mothers are immersed in guilt | 3.1. Increasing daughters’ risk of cancer | 17 (80.9%) |
|  | 3.2. Influencing daughters’ development | 21 (100.0%) |
|  | 3.3. Imposing burdens on daughters | 18 (85.1%) |
